# Vocalizations of the Pekin duck (*Anas platyrhynchos domesticus*): how stimuli, sex, and social groups affect their vocal repertoire

**DOI:** 10.1016/j.psj.2024.103738

**Published:** 2024-04-05

**Authors:** J.M. Schober, J. Merritt, M. Ulrey, T.Y. Yap, J.R. Lucas, G.S. Fraley

**Affiliations:** ⁎ Animal Sciences, Purdue University, West Lafayette, IN, USA; † Biology Department, Purdue University, West Lafayette, IN, USA

**Keywords:** Pekin duck, vocalization, repertoire

## Abstract

Pekin ducks are exposed to stressors such as heat stress, enteric pathogens, mycotoxins, and other environmental stressors. We know from wild bird literature that birds communicate through vocalizations. We hypothesized that Pekin ducks would have a diverse repertoire that is affected by the sex, social group, and specific stimuli. We utilized adult Pekin ducks to develop a vocal repertoire. We placed 1 to 4 ducks of varying sexes into a sound chamber with various stimuli used to encourage new vocalizations. Birds were recorded for 20 min with several variations of number and sexes of ducks. Once the ducks were recorded each vocalization that was clipped was named based on a predetermined naming system. We characterized the vocal system of the ducks under each stimulus and social treatment in 4 ways: overall call rates, call diversity, call repertoire, and call spectral properties. In all cases, normality of residuals and homogeneity of variances for GLM and ANOVA models were confirmed using Proc Univariate (SAS v9.4) where a *p* ≤ 0.05 was considered significant. We found that Pekin ducks produce up to 16 different vocalizations. The treatments had a significant effect on the overall rate of calls given by the ducks (ANOVA: F_6,31_ = 8.55, *p* < 0.0001). Ducks produced the most calls by far when someone was sitting in the chamber with them (30.04 ± 4.45 calls/min). For call diversity, we found that there was a significant main effect of hen number (F_218_ = 12.21, *p* = 0.0004) but no main effect of drake number (F_3,18_ = 3.04, *p* = 0.0555). Cluster analyses indicated that certain types of calls were given under specific conditions. There were generally 6 major clusters of vocal repertoires (R-square = 0.899, Cubic Clustering Criterion = 9.30). Our results suggest that Pekin ducks are affected by the types of stimuli and social environment in how much they vocalize and in the properties of the calls they use. In addition, males and females differ somewhat in the repertoire of the calls they use, and in the spectral properties of their calls.

## INTRODUCTION

The Pekin duck was domesticated from the Mallard duck between 4,000 and 10,000 years ago and is the predominant meat type duck in the world (Cherry and Morris, 2008). The global production of waterfowl is a rapidly growing industry. Total meat duck production increased from 2.9 million tons in 2000 to nearly 4.4 million tons in 2013, a growth rate of 3.2% and has further increased to 7.2 million tons in 2018 (Chen et al., 2021). The USA is the third largest producer of duck products in the world, producing nearly 36 million ducks annually (Chen et al., 2021).

With an increase in production, we are increasing the metabolic and physical demands on these ducks. In addition, animal welfare concerns are becoming predominant and an important aspect of raising production animals, thus, we need to develop methodologies for noninvasive and real-time assessment of flock welfare in order to provide our animals with a stress-free environment. Unfortunately, like all poultry, flocks of Pekin ducks are exposed to stressors such as heat stress, enteric pathogens, mycotoxins, and other environmental stressors. Even regular management practices such as daily egg collection, vaccinations, or bedding placement can be a source of stress (Cherry and Morris, 2008; Chen et al., 2021).

All of our poultry species are social animals, and this is particularly true for the Pekin duck (Cherry and Morris, 2008). Social behaviors are equally important in the Mallard, and vocalizations have been shown to be critical components of their interactions and behaviors (Abraham, 1975; Hicinbothom and Miller, 2018). Indeed, animal communication is a key element of social behavior across the entire animal kingdom (Bradbury and Vehrencamp, 2011). For example, specific types of maternal vocalizations, or calls, can alter Mallard behaviors (Miller and Gottlieb, 1978; Blaich et al., 1988; Hicinbothom and Miller, 2018) and some Mallard calls have even been shown to affect conspecifics’ heart rates and physiology (Evans and Gaioni, 1990; Thompson et al., 1968). Despite this knowledge of wild Mallards, poultry scientists have yet to take the natural and investable next step to utilize flock vocalizations as indicators of welfare status.

Vocalizations can also be used to determine if an animal is experiencing a specific stimulus. Birds have many species-specific songs and calls. Calls are vocalizations that are produced by all birds, at all ages, during all times of the year for a variety of functions (Ballentine and Hyman, 2021). Examples of different types of calls include begging signals, contact calls, migratory flight calls, food calls, and alarm calls (Ballentine and Hyman, 2021). Alarm calls are key antipredator strategies, and different types of alarm calls are categorized by the context in which they are given, and the level or type of predatory threats that exist, but also to some degree by the way they sound (Caro, 2005; Ballentine and Hyman, 2021). For example, seet alarm calls are high-frequency, narrow-bandwidth calls, typically given by small birds when they detect an avian predator in flight that may be actively hunting for prey (Ballentine and Hyman, 2021). Mobbing alarm calls are typically given when a bird has spotted a terrestrial or perched predator. These calls can be short, simple “chip” notes or longer broad band sounds that could be described as harsh, rough, or raspy. These calls attract members of the same species (conspecifics) and members of different species (heterospecifics) to elicit a behavior known as mobbing (Caro, 2005; Kalb and Randler, 2019). Distress alarm calls are given in the most extreme situation – when an animal has been attacked or captured by a predator (Ballentine and Hyman, 2021). Distress calls tend to be loud and harsh, broad-frequency sounds that could be described as a “scream” (Magrath et al., 2015; Ballentine and Hyman, 2021). Distress calls are not meant to cause others to dive for cover, but often attract parents, other family members, or flock mates, or even members of different species. Contact calls allow birds to coordinate movements of a group and recognize preferred social partners. Migratory flight calls are produced by many migratory birds at night during their flights, though the function of these calls is unknown (Ballentine and Hyman, 2021). Thus, vocalizations in birds provide a great deal of information about their status and welfare.

A variety of calls are commonly observed in wild birds, but no one has recorded and analyzed the full vocal repertoire of the Pekin duck. Abraham (1975) explored and extended the qualitative descriptions of the vocal displays in wild Mallards and Ferreira et al. (2022) found that chickens under a commercial-like setting were unable to discriminate between alarm calls and contentment calls; however, no one has characterized the entire vocalization repertoire in any poultry species. Collias (1987) attempted to classify the vocal repertoire of the Red Junglefowl, which is the wild ancestor of the domestic chicken. The research was conducted in a zoo setting, and 24 vocal signals were described, but no absolute size of the vocal repertoire could be determined as some of the vocal signals can be intergradation between some signals and between different stimuli (Collias, 1987). The current research used updated technology and a controlled environment while using different stimuli and social groups to elicit a relatively complete repertoire of Pekin ducks.

With little research on this topic, a better understanding of the birds’ calls could help us to better understand their welfare and wellbeing, from their point of view (Clucas et al., 2004; Gall et al., 2012; Ronald et al., 2015; Sun et al., 2021). We know from the wild bird literature that birds communicate with each other in various ways, one way being through vocalizations (Saunders, 1941; Catchpole and Slater, 2008; Bradbury and Vehrencamp, 2011). Since Pekin ducks are domesticated Mallard ducks, we hypothesized that Pekin ducks would have a diverse repertoire that is affected by the sex, social group, and specific stimuli. We chose to work with the Pekin duck as it is the most common commercial breed globally and the duck is the second most common food animal globally.

## MATERIALS AND METHODS

### Animals

A total of 29 Pekin ducks from 35 wk of age at the start of the experiment to 45 wk of age at the end of the experiment were studied. The ducks were housed following industry standards, with ad lib water and feed provided 8 h per day (Chen et al., 2021). Ducks were fed standard breeder diet (Oluwagbenga et al., 2022; Tetel et al., 2022a,b). They were placed in a single room with an 18:6 light cycle, and temperature of 20 to 22°C. Water nipple lines (5 ducks per nipple) were placed over a pit covered with plastic flooring, and the remaining area of the room was covered with shavings and added to or replaced as necessary. All procedures were approved by Purdue's Institutional Animal Care and Use Committee (PACUC # 2109002194).

### Anechoic Chamber

**Figure 1:**
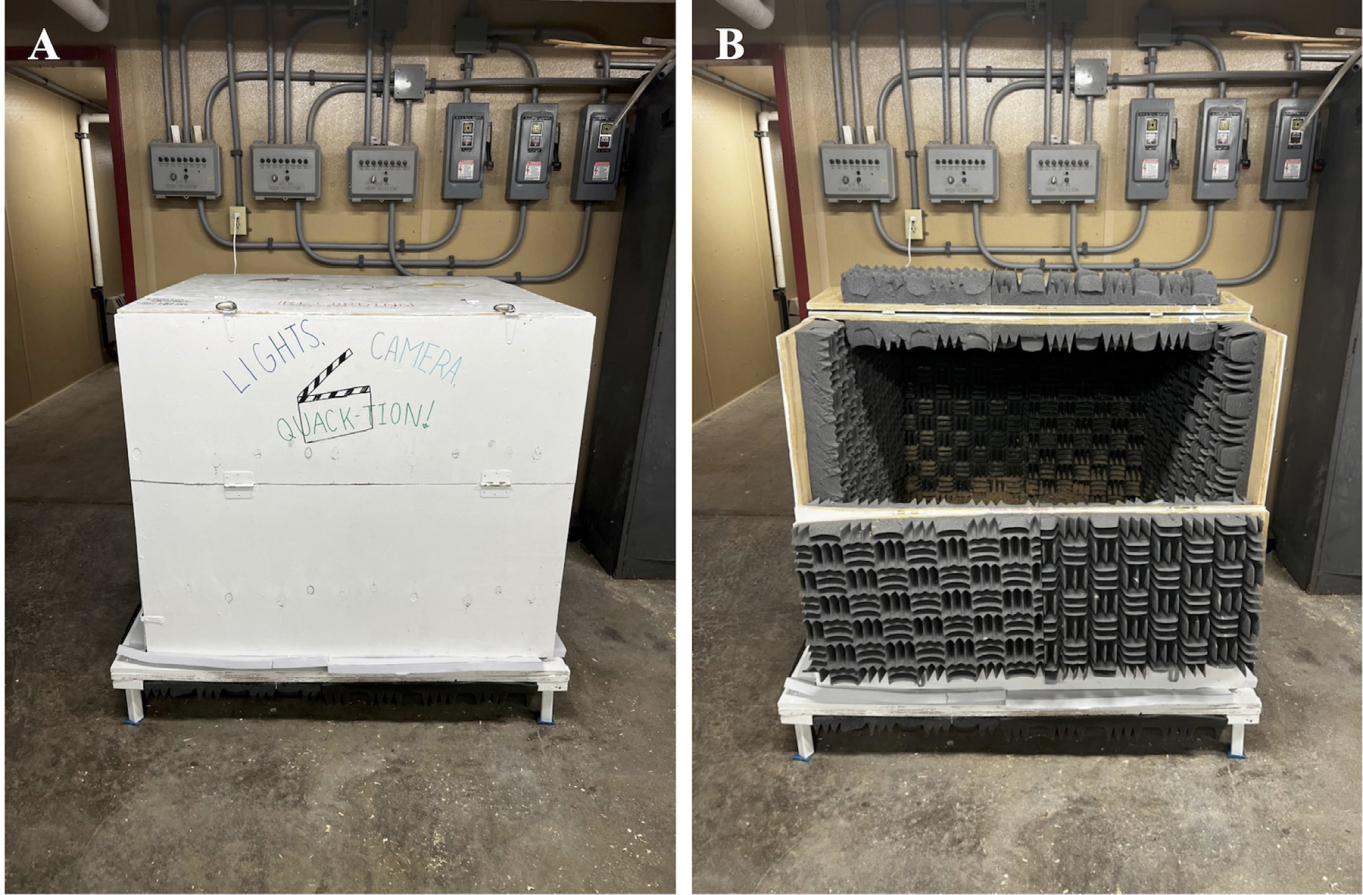
Anechoic chamber used to record duck vocalizations. Made out of plywood (A) lined with anechoic foam (B; Classic 3″ foam (UNX-3), attached with acoustic adhesive (PA-04; Memtech Acoustical, Rochester Hills, MI) to the inside walls to minimize echoes and reverberations from the barn or chamber. Shavings were placed at the bottom to provide a sense of normalcy to the ducks while in the chamber.

Vocalizations were recorded in an anechoic chamber to reduce background noise. The anechoic chamber was a plywood box lined with anechoic foam (Classic 3″ foam (UNX-3), attached with acoustic adhesive (PA-04; Memtech Acoustical, Rochester Hills, MI) to the inside walls to minimize echoes and reverberations from the barn or chamber (Figure 1). We used a Zoom H5 recorder (Zoom North America, Hauppauge, NY), an Audio Technica AT4022 omnidirectional microphone (Audio-Technica, Stow, OH) and a WYZE Cam v3 Bluetooth camera (Wyze Labs, Kirkland, WA) to record the ducks’ vocalizations and behaviors in the chamber. The anechoic chamber was used as a novel environment and was not a representative of their environments in a commercial or research setting, as it was meant to elicit as many vocalizations as possible. The anechoic chamber was also only used for short periods of recordings in order to not decrease the ducks’ welfare.

### Stimuli

We placed a range of 1 to 4 ducks of varying sexes and social groups into the chamber for approximately 45 min. The social groups included different numbers of drakes and hens, and different individuals of each sex. Different stimuli were used to encourage new vocalizations potentially associated with positive or negative affective states. These stimuli included a remote-controlled toy dragon (**DR**), ball with zip ties attached (**DT**; Colton and Fraley, 2014), a preening cup (**PC**; semi-open water; Schober et al., 2023), LED lights (**LD**), black lights (**BL**), a researcher sitting in the chamber with them (**ST**) and no stimuli (**NO**). Birds exposed to each stimulus were recorded for 20 to 30 min with several variations of number and sexes of ducks. A total of 4 hens and 4 drakes were exposed to the BL, LD, PC, and DR stimuli, 4 drakes and 5 hens were exposed to the DT stimuli, 5 drakes and 4 hens were exposed to NO, and 2 drakes and 2 hens were exposed to the ST stimuli (Table 1). We placed the first duck into the chamber and immediately put in the stimulus. After ∼8 min, we then placed the second duck into the chamber with the first duck and the stimulus and repeated this process for the different treatments/different numbers/types of social groups.

**Table 1:** Number of hens and drakes exposed to each stimulus.

Treatments	Drakes	Hens
Black light	4	4
Ball with zip ties	4	5
Researcher sit-in	2	2
LED lights	4	4
Preening cup	4	4
Dragon	4	4
No stimuli	5	4

### Analysis of Audio Recordings

Once the ducks were recorded in the sound chamber, we analyzed their vocalizations via the use of Adobe Premiere Pro, Adobe Audition, and Praat. Adobe Premiere Pro is a video editing program that allowed us to stitch together videos and audio recordings from the anechoic chamber. We then used Adobe Audition, a digital audio workstation with a waveform editing view used to isolate each vocalization. We also used Praat (Boersma 2001), a phonetics software, in conjunction with Adobe Audition, as it gave us a different view of each vocalization so we could correctly characterize and name each call (see Lucas et al. 2015). Each vocalization that was clipped was named based on a pre-determined naming system. This naming system was created based on the following criteria: number of pulses (number of waves when looking at a wavelength), amplitude (how loud each sound is, measured in decibels, [**dB**]), frequency (rate of the vibration of the sound traveling through the air, measured in hertz [**Hz**]), and the shape of any frequency modulation (variation of the frequency within a portion of the vocalization).

### Statistical Analyses

We characterized the vocal system of the ducks under each stimulus and social treatment in 4 ways: overall call rates, call diversity, call repertoire, and call spectral properties (i.e., the frequency and amplitude modulation properties of each call). In all cases, normality of residuals and homogeneity of variances for GLM and ANOVA models were confirmed using Proc Univariate (SAS v9.4).

*Overall Call Rate Analysis.* Every treatment had a different combination of individuals; therefore, we did not run repeated measures ANOVA for overall call rate. We instead ran an ANOVA where *p* ≤ 0.05 was considered significant. The call rates were calculated as the total number of calls of any type given by birds per minute in each trial. Thus, each trial generated a single call rate.

*Call Diversity*. Call diversity for each social and stimulus treatment was calculated using Shannon Entropy (H; see Freeberg and Lucas 2012):

$$\text{H}=\overset{\;n}{\underset{\;i=1}{Sp_{i}\left(-\text{lo}g_{2}p_{i}\right)}}$$

where pi = the proportion of each of n call types; n = the total number of call types – here 16;

H = Shannon index measured in bits.

Shannon Entropy measures are affected by the overall sample size because the maximum Entropy is:

$$H_{\text{max}}=\text{lo}g_{2}N$$

Where N = sample size.

To account for this, we report a Shannon Entropy Index as the Shannon Entropy as a fraction of the maximum Entropy based on the total number of calls given in a given trial:

$$H_{i}=H/\text{Hmax}$$

*Call Repertoire*. The call rates of each social group under each stimulus were calculated as the number of each call type recorded for that trial divided by the duration of the trial. This set of call-specific rates is a vector describing how the birds responded vocally to the treatment. We ran the vectors through a cluster analysis (Proc Cluster; SAS v9.4) and plotted out the tree (Proc Tree; SAS v9.4). The number of significant clusters was determined based on the maximum Cubic Clustering Criterion with 15 potential clusters.

*Call Spectral Properties.* The spectral properties of each call were analyzed using warblerR code (see Keen et al. 2021) to estimate 68 spectral properties of each call given by ducks in each social and stimulus environment (Table 2).

**Table 2:** Measures of acoustic structure. Spectrographic parameters used in the canonical discriminant analysis. The first column indicates the parameters, and the second column indicates the function used in the warblerR code.

Measure of acoustic structure	
Time and frequency	duration, meanfreq, sf
Frequency amplitude distribution	freq.median, freq.Q25, freq.Q75, freq.IQR, sp.ent, peakf, meanpeakf
Distribution of amplitude in time	time.median, time.Q25, time.Q75, time.IQR, skew, kurt, time.ent, entropy, sfm
Fundamental frequency contour descriptors	meanfun, minfun, maxfun
Dominant frequency contour descriptors	meandom, mindom, maxdom, dfrange, modindx, startdom, enddom, dfsslope
Harmonic content descriptors	hn_freq, hn_width, harmonics, HNR
Cepstral coefficients	min.cc1, min.cc2, min.c3, min.cc4, min.cc5, max.cc1, max.cc2, max.cc3, max.cc4, max.cc5, median.cc1, median.cc2, median.cc3, median.cc4, median.cc5, mean.cc1, mean.cc2, mean.cc3, mean.cc4, mean.cc5, var.cc1, var.cc2, var.cc3, var.cc4, var.cc5, skew.cc1, skew.cc2, skew.cc3, skew.cc4, skew.cc5, kurt.cc1, kurt.cc2, kurt.cc3, kurt.cc4, kurt.cc5, mean.d1.cc, var.d1.cc, mean.d2.cc, var.d2.cc

We then ran a canonical discriminant analysis (**CDA**: Proc Candisc, SAS v9.4) to evaluate whether the acoustic properties of the calls used by birds changed across treatments. Here we focus on the first 2 canonical coefficients. For all the AM calls, the first 2 canonical coefficients (can1 and can2) accounted for 0.518 and 0.227 of the total variance. For the pip harm calls, can1 accounted for 0.497 and can2 accounted for 0.1322 of the total variance; for honk calls, can1 accounted for 0.513, and can2 accounted for 0.188 of the total variance; for pip calls, can1 accounted for 0.481 and can2 accounted for 0.130. We grouped all the minor calls together (calls that were produced less than 300 times) and ran a CDA on the entire set of minor calls. Can1 accounted for 0.301 and can2 accounted for 0.169 for these calls. Note that canonical discriminant analysis is a dimension-reduction technique that provides canonical coefficients that are analogous to principal component axes. This approach is valuable because it could indicate that the production of any given call type reflects the physiological response of the ducks to their environment. P ≤ 0.05 was considered significant.

## RESULTS

**Figure 2:**
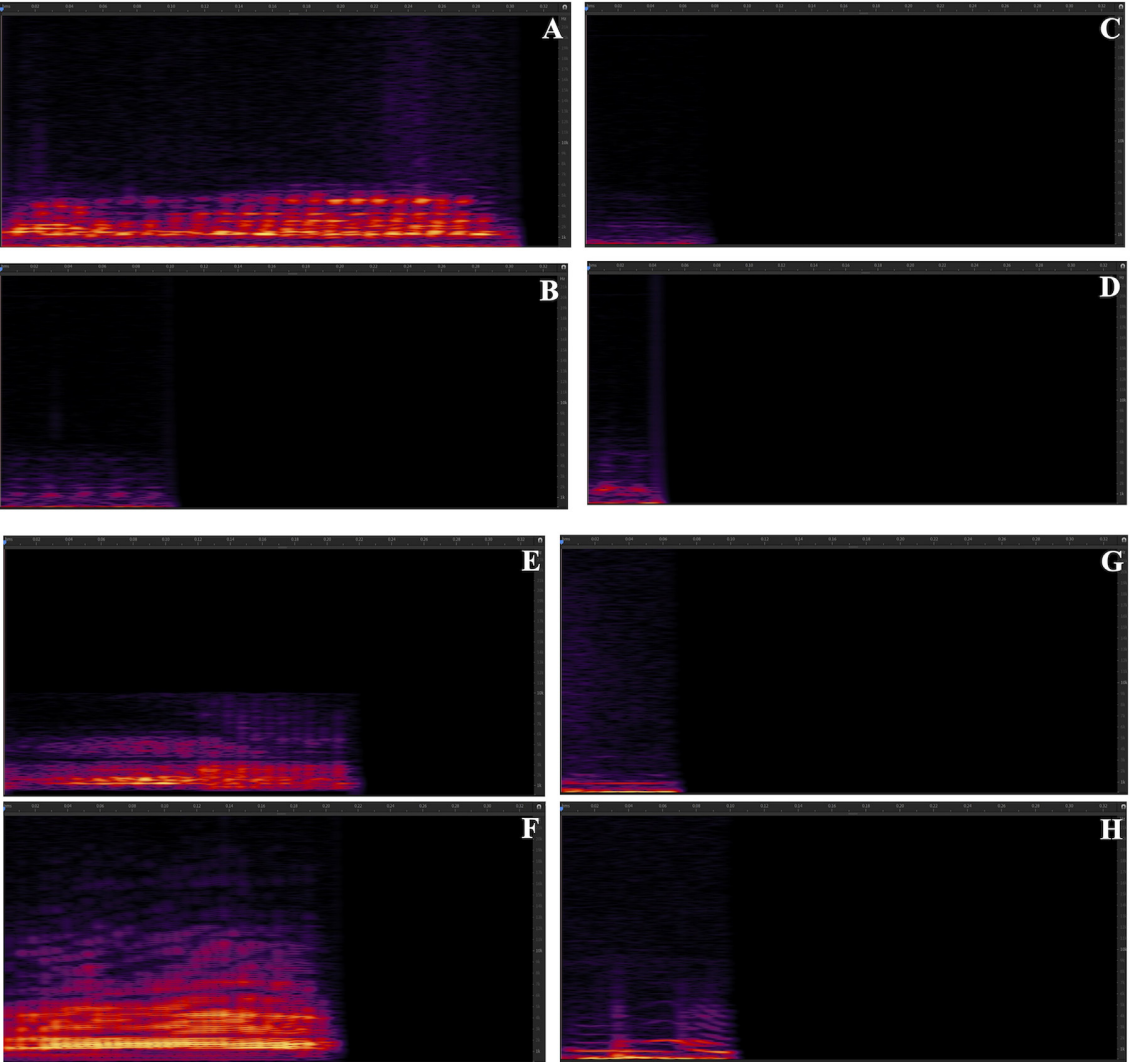

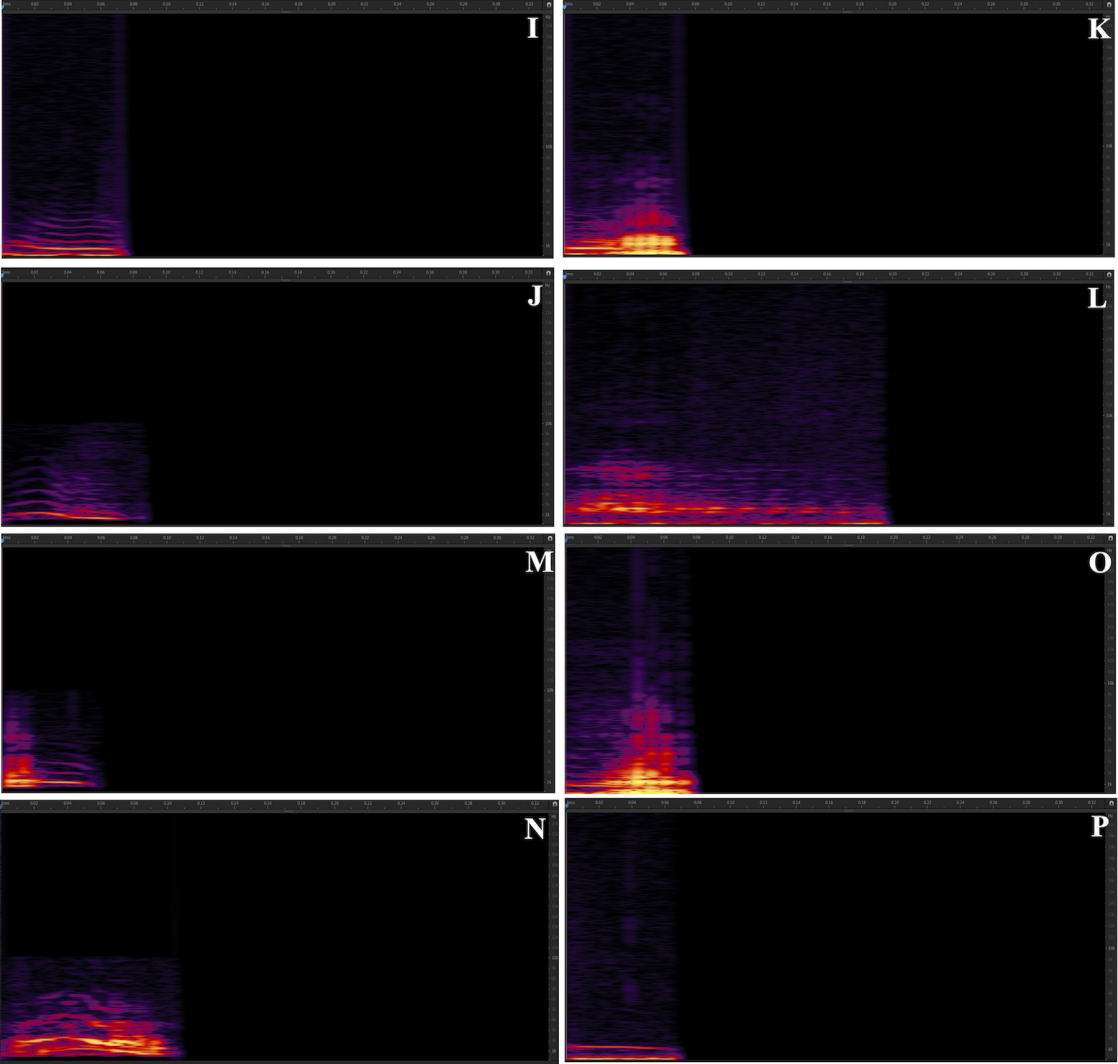
Spectrograms of all recorded vocalizations made by Pekin ducks. (A) AM long; (B) AM medium; (C) AM short (D) AM very short (E) AM honk; (F) honk; (G) pip; (H) pip harmonic; (I) harmonic; (J) harmonic + AM; (K) harmonic honk; (L) honk + AM; (M) honk harmonic; (N) pip + AM; (O) pip honk; (P) pure tone. The X axis is Time (seconds), and the Y axis is Frequency (Hz). All spectrograms have been standardized to start at 0:00:000 and end at 0:00:328 sec.

Our results showed that Pekin ducks produce up to 16 different vocalizations. Particularly common calls included AM, pip, pip-harm, honk, and honk-AM. Figure 2 includes spectrograms of all the vocalizations recorded.

### Overall Call Rates

The treatments had a significant effect on the overall rate of calls given by the ducks (ANOVA: F_6,31_ = 8.55, *p* < 0.0001): ducks produced more calls with the BL compared to the DR (11.00 ± 3.55 calls/min), DT (10.94 ± 3.13 calls/min), no stimuli (11.80 ± 3.49, calls/min), and PC (9.95 ± 3.51 calls/min). The ducks produced the most calls by far when someone was sitting in the chamber with them (30.04 ± 4.45 calls/min).

### Call Diversity

Interestingly, the diversity of the vocal repertoire ducks used (measured using the Shannon Entropy Index, H_i_) changed with the social structure of the flock. There was a significant main effect of hen number (F_218_ = 12.21, *p* = 0.0004) but no main effect of drake number (F_3,18_ = 3.04, *p* = 0.0555). However, there was also a significant interaction between hen and drake number (F_2,18_ = 5.60, *p* = 0.0128). Generally, flocks with no hens showed lower repertoire diversity than those with 1 or 2 hens (Figure 4). In addition, vocal diversity was particularly high with 2 hens and 2 drakes (Figure 4). The implication is that more social complexity increases the amount of information ducks convey to each other, but only when the social group includes hens.

### Call Repertoire

**Figure 3:**
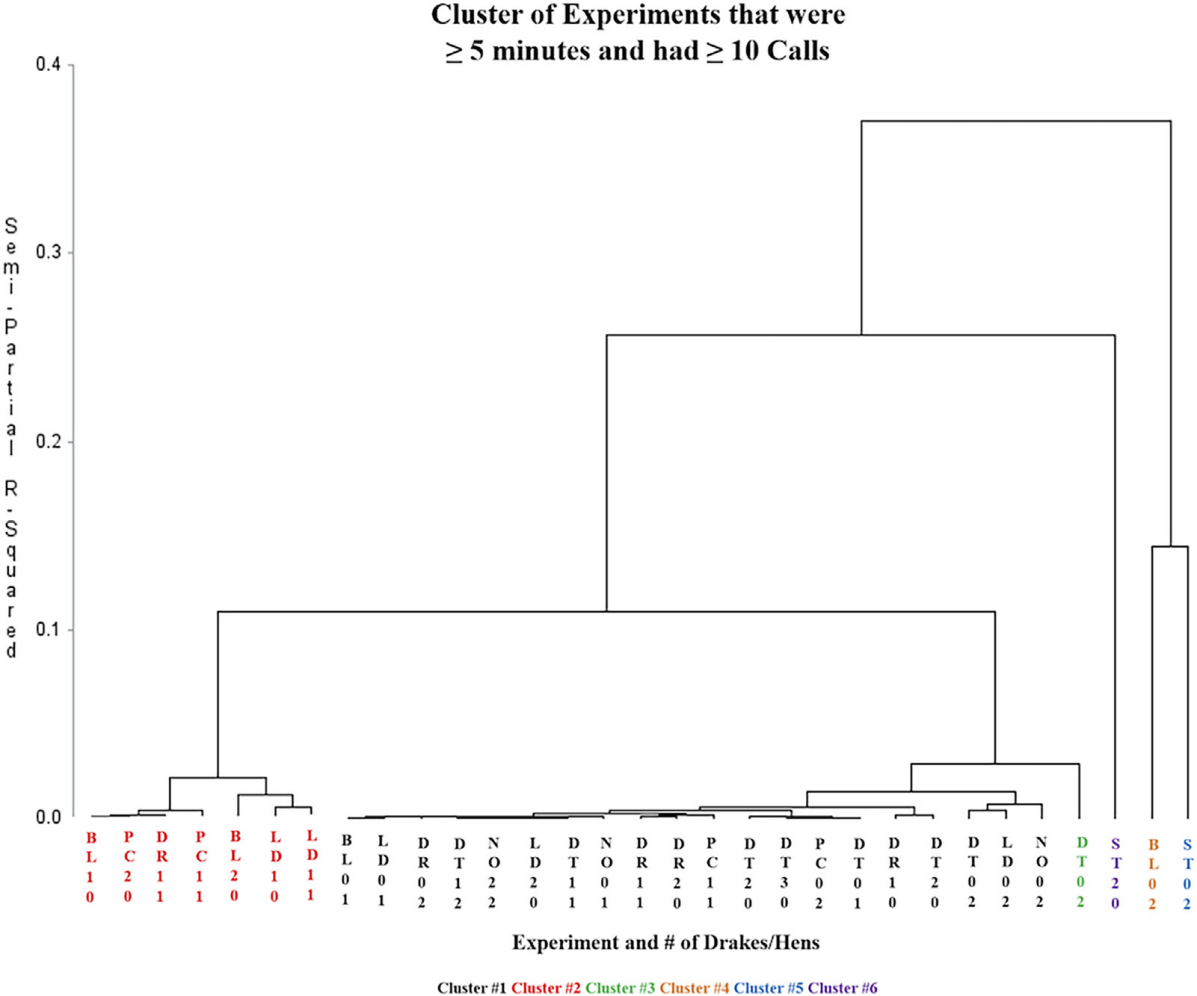
Cluster analysis of experiments that were at least 5 min long and contained at least 10 calls. See key for identification of the 6 main clusters.

The cluster analysis indicated that certain types of calls were given by ducks under specific conditions. There were generally 6 major clusters of vocal repertoires (R-square = 0.899, Cubic Clustering Criterion = 9.30; Figure 3). The main cluster represented 20 different treatments with a variety of stimulus stimuli and social groups; the 2nd cluster included 7 treatments with a variety of stimuli and social groups, but every treatment in this cluster has at least one male; the 3rd cluster was the DT stimulus with only 2 hens; the 4th cluster was the BL stimulus with only 2 hens; the 5th cluster was the ST stimulus with only 2 hens; the 6th cluster was ST with only 2 drakes. These clusters tell us that the ducks’ repertoire is significantly different when 2 hens and no drakes are under BL and DT, and when 2 hens or 2 drakes are in the chamber with researchers, and also when there is at least one drake in the chamber during the BL, PC, DR, or LD stimuli.

**Figure 4:**
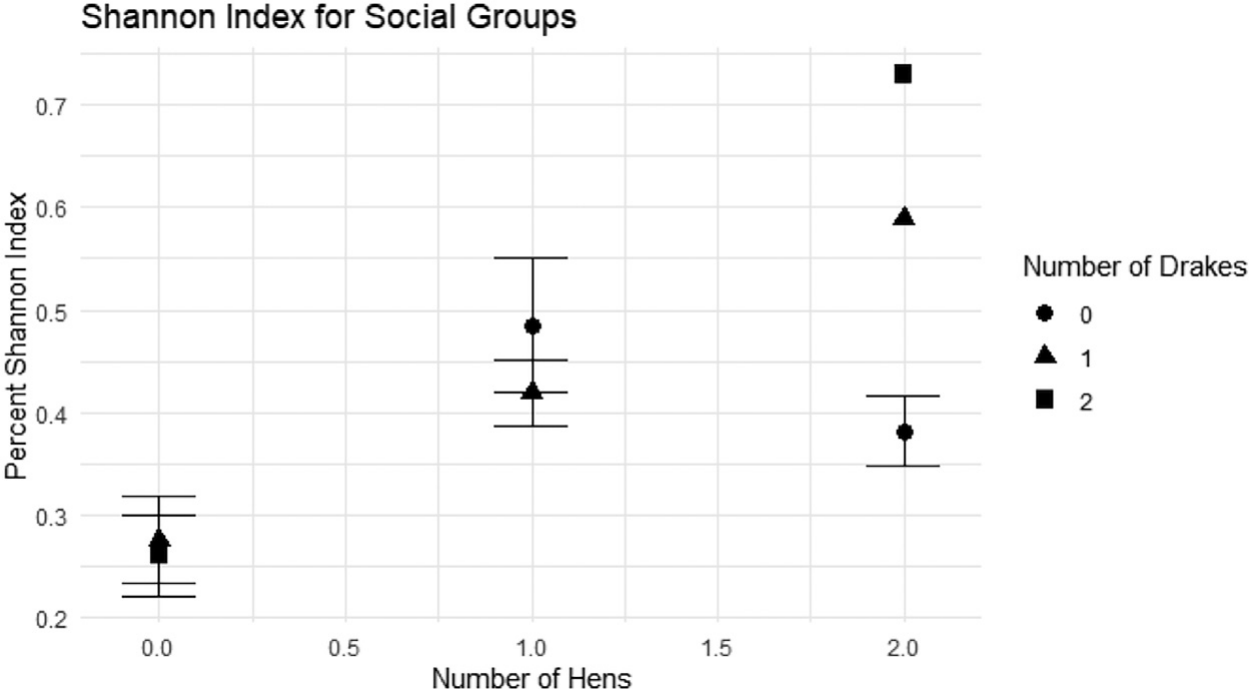
Shannon index for social groups. Number of hens on the X-axis and percent Shannon index on the Y-axis.

These clusters are also able to tell us how social and environmental stimuli affect the rate at which ducks give specific calls. In the 2nd cluster, all of the observations had vocalized the AM long, AM short, and AM very short. In the 3rd cluster, the ducks gave the harm call at a rate of 4.29, and the pip call at a rate of 4.71. In the 4th cluster, the ducks gave the pip call at a rate of 8.00, the pip harm call at a rate of 16.89, the harm + AM call at a rate of 3.02, and the squiggle harm call at the rate of 5.96. In the 5th cluster, the ducks gave the pip call at a rate of 6.86, the pip harmonic call at the rate of 6.10. and the honk call at the rate of 14.29. In the 6th cluster, the ducks gave the AM medium call at the rate of 5,71, the AM short call at the rate of 17.33, and the AM very short call at the rate of 6.29.

### Call Spectral Properties

**Figure 5:**
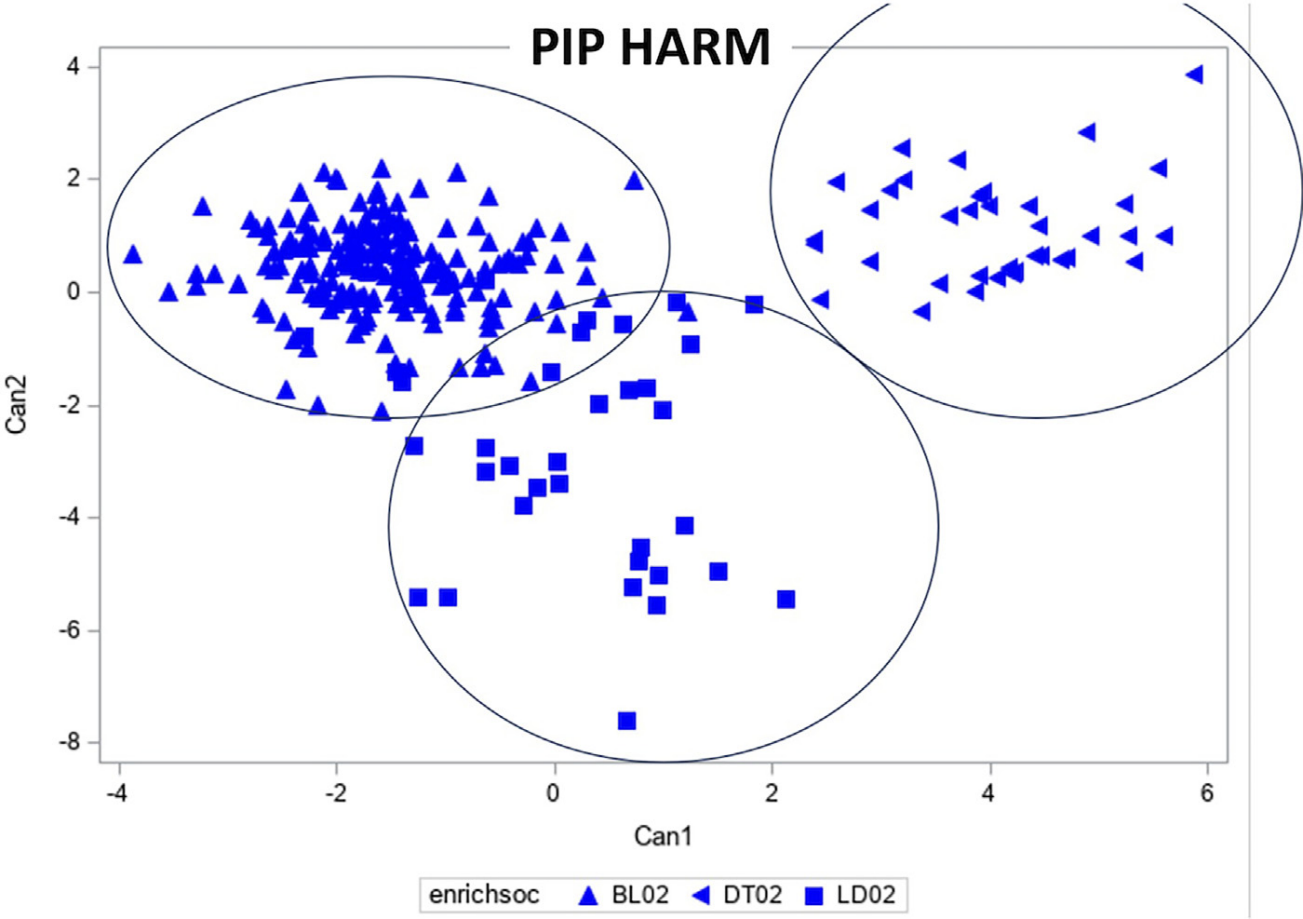
Canonical discriminant analysis of the pip-harm call. Each symbol represents a different stimulus. See key.

*Pip Harm Vocalization.* Only females were recorded giving Pip Harm calls. Moreover, we recorded the most Pip Harm calls from flocks of 2 females – these results are illustrated in Figure 5. The canonical discriminant analysis showed that there was a significant effect of stimulus on Pip Harm spectral properties for the first canonical coefficient (explaining 49.7% of variance, eigenvalue=4.234; F_5, 6.7_ = 6.68, *p* = 0.0152), although there was no significant social group effect (F_2, 7.0_ = 0.44, *p* = 0.659). The second canonical coefficient (explaining 12.5% of variance, eigenvalue=1.125) was squared ((can2+8)**2) to normalize the variance. Neither the stimulus effect (F_5, 6.6_ = 3.25, *p* = 0.0832), nor the social group effect (F_2, 7.3_ = 0.98, *p* = 0.420) were significant. Tables describing the statistics for the first 3 canonical coefficients and the total canonical structure for the canonical discriminant analysis of spectral properties of the Pip-Harm call are located in the supplementary data file (Tables 3 and 4).

**Figure 6:**
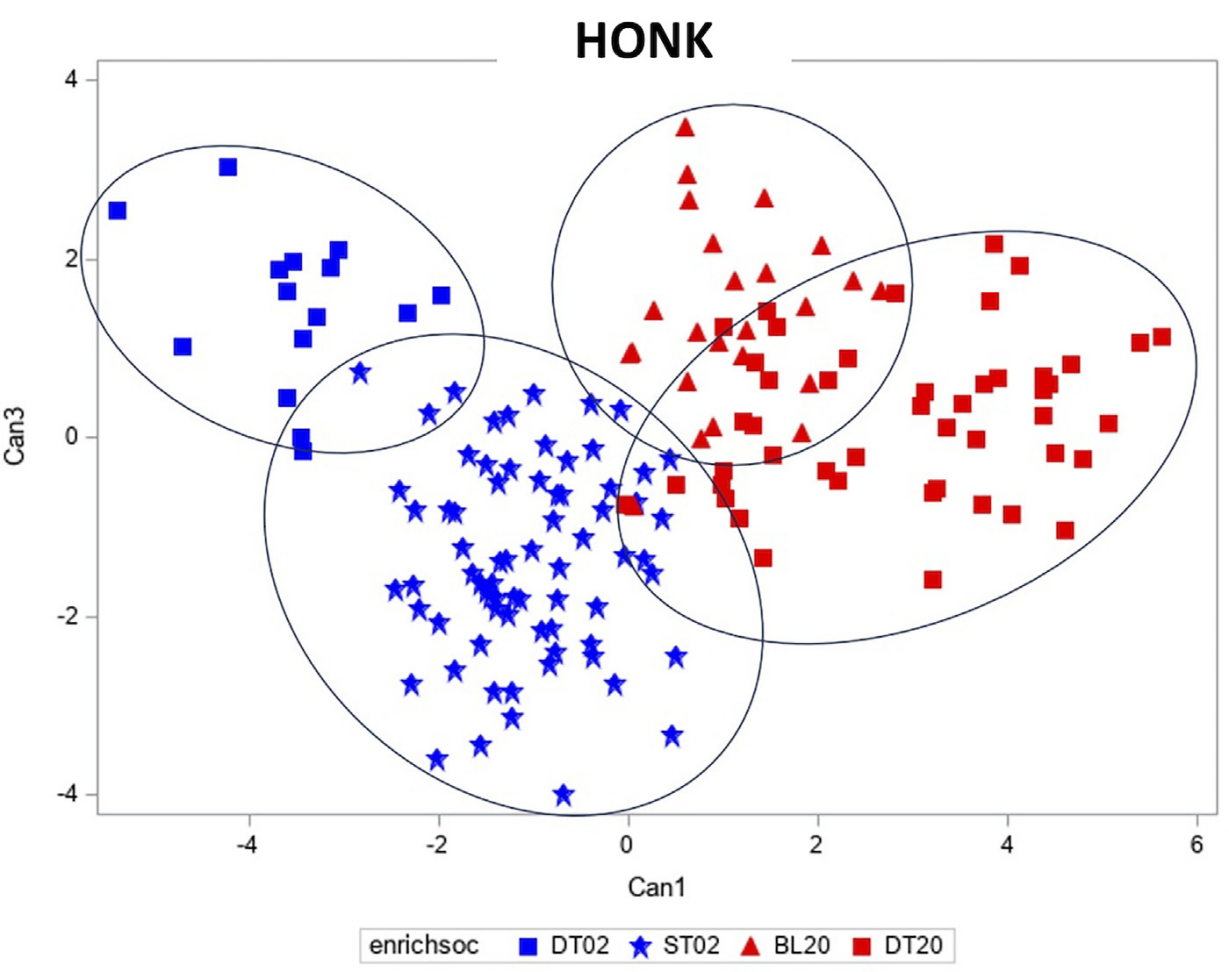
Canonical discriminant analysis of the honk call. Each symbol represents a different stimulus and social group. See key.

*Honk Vocalization*. Both sexes produced Honk vocalizations, with the greatest Honk rates from social groups of either only 2 females or only 2 males. The first canonical coefficient for spectral properties of the Honk calls (explaining 27;7% of variance, eigenvalue = 3.833) was significantly different between sexes (F_6, 20.5_ = 5.36, *p* = 0.0018), although there was no significant stimulus effect for the first canonical coefficient (F_6, 19.5_ = 1.55, *p* = 0.216). The canonical coefficients for the most common calls (DT 2 males, DT 2 females, BL 2 males, ST 2 females) are illustrated in Figure 6.

In contrast, the second canonical coefficient (explaining 12.1% of variance, eigenvalue = 1.678) was significantly different between stimuli (F_6, 19.6_ = 3.17, *p* = 0.0242), but not significantly different across social groups (F_6, 19.6_ = 1.44, *p* = 0.245; Figure 6). The results for the third canonical coefficient (explaining 9.6% of variance, eigenvalue=1.327) were similar: there was a significant effect of stimulus (F_6, 20.3_ = 2.80, *p* = 0.0376) but no effect of social group (F_6, 23.2_ = 0.70, *p* = 0.651) on the third canonical coefficient. Together these results suggest that there is a sex-specific signature in the Honk call, in addition to spectrally different Honk calls being given under different stimuli. Tables describing the statistics for the first 3 canonical coefficients and the total canonical structure for the canonical discriminant analysis of spectral properties of the Pip-Harm call are located in the supplementary data file (Tables 5 and 6).

**Figure 7:**
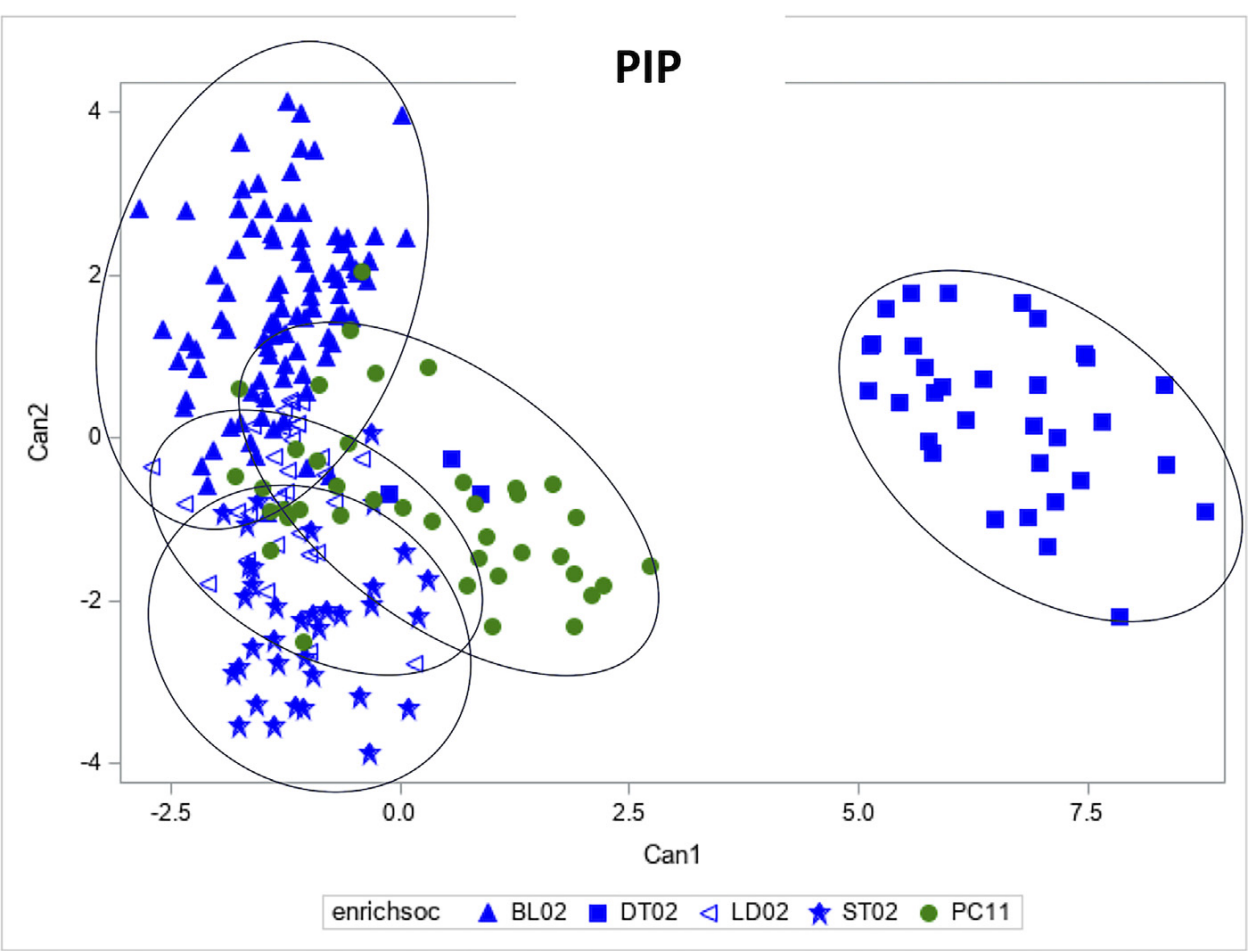
Canonical discriminant analysis of the pip call. Each symbol represents a different stimulus and social group. See key.

*Pip Vocalization.* Almost no Pip calls were given when there were only males in the anechoic chamber. We recorded the most Pip calls from flocks with either 2 females (BL, DT, LD, ST) or a flock of 1 female and 1 male (PC) – these results are illustrated in Figure 7. The canonical discriminant analysis showed that there was a significant effect of stimulus on Pip spectral properties for the first canonical coefficient (explaining 48.1% of variance, eigenvalue=6.751; F_5, 8.9_ = 4.81, *p* = 0.02062), although there was no significant social group effect (F_4, 9.7_ = 0.52, *p* = 0.723). Neither the stimulus effect (F_5, 6.6_ = 3.25, *p* = 0.0832), nor the social group effect (F_2, 7.3_ = 0.98, *p* = 0.420) were significant for the second canonical coefficient (explaining 13% of variance, eigenvalue = 1.819).

## DISCUSSION

Vocalizations in wild birds have been studied for centuries (Mathews, 1904; Saunders, 1941). This entire field of biology, along with all of its tools to analyze vocalizations in birds, has been virtually ignored by poultry science despite the potential relevance they may have to the commercial industry. Vocalizations as an indicator of stress have been used in many production animals, including cattle, sheep, pigs, horses, and goats (Kiley, 1972; Liebenberg et al., 1977; Klingholz et al., 1979; Hall, 1989; Grauvogl, 1948; Manteuffel et al., 2004). However, there has been very limited research conducted on the vocalizations of poultry, and specifically, no research has been conducted on Pekin ducks. Overall, our data imply that there is variation in the physiological response to the different environmental stimuli, and that a spectral analysis of the calls that ducks generate under different conditions can be used as a proxy of those physiological or emotional responses.

Vocalizations can be a great tool to interpret behavior, health, and welfare states of animals (Manteuffel et al., 2004). In the chicken, electrical stimulation of various brain areas can elicit reinforcing or aversive emotions in parallel to vocalization types that are normally produced in respective behavioral and environmental contexts (Andrew, 1969). These observations support the idea that some welfare-relevant emotions are closely related to specific vocalizations. Frightening or unnatural environments, disturbed behavioral homeostasis, and impaired welfare increases distress calls in chickens (Richard-Yris et al., 1998). Distress calls produced by chicks in isolation may signal social distress and search for contact, although it has also been claimed to be a common pattern in the regular vocalization of chicks in non-isolation situations (Schmidt and Marx, 1998).

In wild birds, songs consist of species typical sequences of vocalizations that are learned from adult tutors, refined, and maintained through practice, and then used primarily within reproductive contexts to attract mates and define breeding territories (Catchpole and Slater, 2008; Bradbury and Vehrencamp, 2011). Play behavior in birds (e.g., play fighting, acrobatics, and object manipulation) has been estimated to be present in only 1% of approximately 10,000 bird species (Emery and Clayton, 2015). However, it may be that vocalizing outside of a breeding context is a prevalent form of play exhibited by an estimated 4,500+ species of oscine songbirds (Sibley and Monroe, 1990). Darwin (1871) claimed that “birds continue singing for their own amusement after the season for courtship is over.” Birds who learn song only during a single critical period engage in periods of singing outside reproductive contexts, and depriving birds of this form of song “practice” can result in degraded song structure (Woolley and Rubel, 1997).

Poultry are not songbirds. However, that does not negate the complexity of their vocalization repertoire nor the importance of their vocalizations to express their current emotional status to their conspecifics. A study from Brenowitz (1978) looked at Gila woodpecker vocalizations and found that they have several calls they use when faced with disturbances from humans and other species of birds. Chestnut-crowned Babbler's were shown to have calls that were given when they perceived an imminent, airborne threat; this call caused conspecifics to look upwards while sometimes in combination with either freezing or darting into undergrowth for cover (Crane et al., 2016). Lee at al. (2015) found that physical and mental stress, such as heat stress, handling, or fear, affects laying hen vocalizations. Researchers have discovered 20 to 25 discrete vocalizations in chickens, but the meaning underlying each vocalization is still unknown (Collias and Joos, 1953; Evans and Evans, 1999; Marx et al., 2001).

Our results showed that Pekin ducks produce up to 16 different vocalizations under the conditions we tested in this study. We also identified 4 additional egg laying vocalizations (unpublished observation). Particularly common calls included AM, pip, pip-harmonic, honk, and honk-AM (Figure 1). Our results also suggest that ducks are affected by types of stimuli and social environment in how much they vocalize and in the properties of the calls they use. In addition, males and females differ somewhat in the repertoire of calls they use, and in the spectral properties of their calls.

Overall, our data imply that there is variation in the emotional response to the different environmental stimuli, and that a spectral analysis of the calls that ducks generate under different conditions could be used as a proxy of those emotional, and likely physiological, responses. Future studies should examine the physiological effects these different calls cause in conspecifics. This would help determine which calls are alarm calls, and which calls are calming calls. Knowing the difference between the calls, a device could be created to identify calls caused by specific types of stressors, and thus eliminate the stressor before we observe a drop in production or flock welfare. Future studies should assess the vocal repertoires of other poultry species, such as chickens and turkeys, as well as differences among breeds within these species, as different types of birds produce different types of vocalizations. Future studies should also assess the prolonged exposure to specific stimuli/conditions to see how it affects the vocal patterns of the specific species. Future studies will use this repertoire to train a machine learning program to be able to detect certain vocalizations withing a barn to track stressed/sick/injured ducks.

## Appendix Supplementary materials

Image, application 1

## Footnotes

Supplementary material associated with this article can be found in the online version at doi:10.1016/j.psj.2024.103738.
