# Supplementary material for: Vocalizations of the Pekin duck (Anas platyrhynchos domesticus): how stimuli, sex, and social groups affect their vocal repertoire
Source: Poult Sci. 2024 Apr 5;103(7):103738. doi: 10.1016/j.psj.2024.103738 (PMC11112367; doi:10.1016/j.psj.2024.103738)
Supplement: Supplementary file 1 [file mmc1.docx]

**Supplemental Tables**

**Table 3.** Statistics for first 3 canonical coefficients for the Pekin duck Pip-Harm call

|  | **Canonical Correlation** | **Adjusted Canonical Correlation** | **Approximate Standard Error** |  | | | | **Test of H0: The canonical correlations in the current row and all that follow are zero** | | | | |
| --- | --- | --- | --- | --- | --- | --- | --- | --- | --- | --- | --- | --- |
|  |  |  |  | **Eigenvalue** |  | **Proportion** | **Cumulative** | **Likelihood Ratio** | **Approximate F Value** | **Num DF** | **Den DF** | **Pr > F** |
| **1** | 0.899 | 0.876 | 0.010 | 4.23 |  | 0.49 | 0.497 | 0.0065 | 2.50 | 715 | 3034. | <.0001 |
| **2** | 0.727 | 0.652 | 0.025 | 1.12 |  | 0.13 | 0.629 | 0.0342 | 1.76 | 640 | 2770. | <.0001 |

**Table 4.** Total Canonical Structure for Canonical Discriminant Analysis of spectral properties of Pekin duck Pip-Harm call.

| **Total Canonical Structure** | | |
| --- | --- | --- |
| **Variable** | **Can1** | **Can2** |
| **sd** | 0.149428 | 0.076107 |
| **freq_median** | -0.040471 | 0.134603 |
| **freq_Q25** | -0.154066 | 0.201823 |
| **freq_Q75** | 0.084404 | 0.039478 |
| **freq_IQR** | 0.152462 | -0.020038 |
| **time_median** | 0.140528 | 0.156292 |
| **time_Q25** | 0.216385 | 0.150724 |
| **time_Q75** | 0.126002 | 0.152927 |
| **time_IQR** | -0.042066 | 0.098531 |
| **skew** | 0.231289 | -0.367477 |
| **kurt** | 0.201056 | -0.390215 |
| **sp_ent** | -0.062519 | 0.351310 |
| **time_ent** | -0.087123 | -0.289277 |
| **entropy** | -0.103911 | 0.215910 |
| **sfm** | 0.067956 | 0.251299 |
| **meandom** | -0.269534 | 0.176141 |
| **mindom** | -0.114350 | 0.061742 |
| **maxdom** | -0.042897 | 0.064880 |
| **dfrange** | -0.037698 | 0.061835 |
| **modindx** | 0.025817 | 0.165103 |
| **startdom** | 0.000586 | -0.040157 |
| **enddom** | 0.007530 | -0.101295 |
| **dfslope** | 0.011094 | -0.032929 |
| **meanpeakf** | -0.089114 | 0.025926 |
| **dtw_dim_1** | -0.166170 | 0.226536 |
| **dtw_dim_2** | -0.174784 | -0.019515 |
| **dtw_dim_3** | -0.213912 | 0.098215 |
| **dtw_dim_4** | -0.239574 | 0.085871 |
| **dtw_dim_5** | 0.126933 | -0.057767 |
| **min_cc1** | 0.809363 | -0.224783 |
| **min_cc2** | -0.132337 | -0.252934 |
| **min_cc3** | 0.270112 | -0.046120 |
| **min_cc4** | 0.342898 | -0.438733 |
| **min_cc5** | 0.104414 | -0.199173 |
| **max_cc1** | 0.758668 | 0.072767 |
| **max_cc2** | 0.000081 | 0.029253 |
| **max_cc3** | 0.111314 | 0.201173 |
| **max_cc4** | 0.221260 | -0.313307 |
| **max_cc5** | 0.176131 | -0.379431 |
| **median_cc1** | 0.807479 | -0.109256 |
| **median_cc2** | 0.022664 | -0.087327 |
| **median_cc3** | 0.242299 | 0.034604 |
| **median_cc4** | 0.319765 | -0.455205 |
| **median_cc5** | 0.170114 | -0.207854 |
| **mean_cc1** | 0.828750 | -0.111289 |
| **mean_cc2** | -0.022727 | -0.123414 |
| **mean_cc3** | 0.247098 | 0.053482 |
| **mean_cc4** | 0.341463 | -0.466675 |
| **mean_cc5** | 0.185306 | -0.273908 |
| **var_cc1** | 0.048110 | 0.374447 |
| **var_cc2** | 0.256835 | 0.182954 |
| **var_cc3** | -0.159173 | 0.156729 |
| **var_cc4** | -0.226135 | 0.015108 |
| **var_cc5** | 0.050794 | -0.404274 |
| **skew_cc1** | -0.043957 | 0.160775 |
| **skew_cc2** | -0.101074 | -0.022139 |
| **skew_cc3** | -0.025064 | 0.084378 |
| **skew_cc4** | 0.019193 | 0.070140 |
| **skew_cc5** | 0.002025 | -0.141341 |
| **kurt_cc1** | -0.109129 | -0.009070 |
| **kurt_cc2** | -0.126956 | 0.000291 |
| **kurt_cc3** | -0.082856 | -0.032493 |
| **kurt_cc4** | 0.080012 | -0.057744 |
| **kurt_cc5** | -0.104767 | 0.114052 |
| **mean_d1_cc** | -0.831810 | 0.120005 |
| **var_d1_cc** | -0.778769 | 0.066420 |
| **mean_d2_cc** | 0.765127 | -0.084257 |
| **var_d2_cc** | -0.702406 | -0.13671 |

**Table 5.** Statistics for first 3 canonical coefficients for the Pekin duck Honk call

|  | **Canonical Correlation** | **Adjusted Canonical Correlation** | **Approximate Standard Error** |  | | | | **Test of H0: The canonical correlations in the current row and all that follow are zero** | | | | |
| --- | --- | --- | --- | --- | --- | --- | --- | --- | --- | --- | --- | --- |
|  |  |  |  | **Eigenvalue** | **Proportion** | **Cumulative** | **Likelihood Ratio** | | **Approximate F Value** | **Num DF** | **Den DF** | **Pr > F** |
| **1** | 0.890 | 0.854 | 0.012 | 3.833 | 0.277 | 0.277 | 0.0001 | | 1.73 | 1430 | 4561 | <.0001 |
| **2** | 0.791 | 0.710 | 0.021 | 1.678 | 0.121 | 0.399 | 0.0005 | | 1.47 | 1344 | 4380 | <.0001 |
| **3** | 0.755 | 0.661 | 0.024 | 1.327 | 0.096 | 0.495 | 0.0015 | | 1.34 | 1260 | 4196.6 | <.0001 |

**Table 6.** Total Canonical Structure for Canonical Discriminant Analysis of spectral properties of Pekin duck Honk call.

| **Total Canonical Structure** | | | |
| --- | --- | --- | --- |
| **Variable** | **Can1** | **Can2** | **Can3** |
| **sd** | -0.448685 | 0.221524 | 0.337871 |
| **freq_median** | -0.268868 | 0.022517 | 0.454338 |
| **freq_Q25** | 0.008322 | -0.246377 | 0.324736 |
| **freq_Q75** | -0.206002 | 0.091823 | 0.408221 |
| **freq_IQR** | -0.233509 | 0.168375 | 0.372326 |
| **time_median** | 0.158668 | 0.083437 | -0.146384 |
| **time_Q25** | 0.068884 | 0.123510 | -0.177009 |
| **time_Q75** | 0.212268 | 0.067859 | -0.137284 |
| **time_IQR** | 0.280689 | 0.009168 | -0.076386 |
| **skew** | -0.254645 | 0.302228 | -0.058687 |
| **kurt** | -0.284925 | 0.265506 | -0.005524 |
| **sp_ent** | -0.246678 | -0.028085 | 0.420869 |
| **time_ent** | 0.043170 | -0.068604 | -0.021857 |
| **entropy** | -0.196975 | -0.046908 | 0.344710 |
| **sfm** | -0.382855 | 0.428015 | 0.469127 |
| **meandom** | 0.311766 | -0.364553 | 0.132269 |
| **mindom** | 0.056825 | -0.363422 | 0.113348 |
| **maxdom** | 0.172969 | -0.015614 | -0.002604 |
| **dfrange** | 0.161985 | 0.038073 | -0.019235 |
| **modindx** | 0.182151 | -0.026819 | 0.000655 |
| **startdom** | 0.261599 | -0.427075 | 0.102054 |
| **enddom** | 0.055577 | -0.131233 | 0.064594 |
| **dfslope** | -0.143373 | 0.213712 | -0.040348 |
| **meanpeakf** | 0.333702 | -0.334807 | 0.172433 |
| **dtw_dim_1** | 0.377401 | -0.430255 | 0.121433 |
| **dtw_dim_2** | -0.123577 | 0.219928 | -0.034763 |
| **dtw_dim_3** | -0.079185 | -0.041900 | 0.043272 |
| **dtw_dim_4** | -0.043741 | -0.149147 | 0.099929 |
| **dtw_dim_5** | 0.008252 | 0.053067 | -0.014352 |
| **min_cc1** | 0.639917 | 0.601977 | 0.103707 |
| **min_cc2** | 0.225028 | -0.277145 | -0.320576 |
| **min_cc3** | -0.402781 | 0.563531 | -0.252287 |
| **min_cc4** | -0.269668 | -0.124444 | 0.045632 |
| **min_cc5** | -0.049853 | 0.797694 | 0.230282 |
| **max_cc1** | 0.625734 | 0.578757 | 0.143643 |
| **max_cc2** | 0.278463 | -0.204704 | -0.255142 |
| **max_cc3** | -0.178889 | 0.475264 | -0.249277 |
| **max_cc4** | -0.121409 | -0.272476 | -0.041958 |
| **max_cc5** | 0.069038 | 0.675911 | 0.367402 |
| **median_cc1** | 0.611278 | 0.612068 | 0.128376 |
| **median_cc2** | 0.320094 | -0.261227 | -0.400677 |
| **median_cc3** | -0.301749 | 0.551558 | -0.221332 |
| **median_cc4** | -0.180247 | -0.269351 | -0.058131 |
| **median_cc5** | -0.022980 | 0.781641 | 0.302513 |
| **mean_cc1** | 0.618766 | 0.606606 | 0.125586 |
| **mean_cc2** | 0.314818 | -0.266884 | -0.391937 |
| **mean_cc3** | -0.314872 | 0.562060 | -0.226866 |
| **mean_cc4** | -0.196197 | -0.260999 | -0.054222 |
| **mean_cc5** | -0.017772 | 0.789847 | 0.301866 |
| **var_cc1** | -0.181141 | -0.113065 | 0.218297 |
| **var_cc2** | -0.018319 | 0.108628 | 0.227691 |
| **var_cc3** | 0.303808 | -0.196401 | 0.118651 |
| **var_cc4** | 0.078269 | -0.080037 | -0.014213 |
| **var_cc5** | 0.119051 | -0.264722 | 0.139575 |
| **skew_cc1** | 0.278196 | -0.297707 | 0.049317 |
| **skew_cc2** | -0.123637 | 0.066878 | 0.347374 |
| **skew_cc3** | -0.101531 | 0.079230 | -0.217459 |
| **skew_cc4** | -0.047827 | 0.054242 | 0.196545 |
| **skew_cc5** | 0.084481 | -0.153318 | -0.072006 |
| **kurt_cc1** | -0.167065 | 0.072416 | -0.082813 |
| **kurt_cc2** | -0.157021 | -0.015955 | -0.250732 |
| **kurt_cc3** | -0.048905 | 0.029673 | -0.013893 |
| **kurt_cc4** | -0.041891 | -0.109152 | -0.092997 |
| **kurt_cc5** | -0.025921 | 0.023853 | -0.026930 |
| **mean_d1_cc** | -0.611952 | -0.605600 | -0.124130 |
| **var_d1_cc** | 0.666820 | 0.269780 | -0.097999 |
| **mean_d2_cc** | 0.662683 | 0.578627 | 0.114452 |
| **var_d2_cc** | 0.589282 | 0.252028 | -0.168106 |
|  |  |  |  |
